# Baseline levels and dynamic changes of cfDNA, tumor fraction and mutations to anticipate the clinical course of small cell lung cancer (SCLC) patients treated with first-line atezolizumab and chemotherapy: an hypothesis generating study (CATS/ML43257)

**DOI:** 10.1186/s13046-025-03434-3

**Published:** 2025-06-19

**Authors:** Giulia Pasello, Giulia Pigato, Daniela Scattolin, Stefania Lando, Sara Potente, Chiara Romualdi, Anna Roma, Maria Vittoria Resi, Stefano Frega, Alessandra Ferro, Alessandro Dal Maso, Laura Bonanno, Valentina Guarneri, Elisabetta Lazzarini, Stefano Indraccolo

**Affiliations:** 1 Department of Surgery, Oncology and Gastroenterology, University of Padova, Padova, Italy; 2 Medical Oncology 2, Veneto Institute of Oncology IOV-IRCCS, Padova, Italy; 3 Basic and Translational Oncology Unit, Veneto Institute of Oncology IOV-IRCCS, Padova, Italy; 4 Unit of Biostatistics, Epidemiology and Public Health, Department of Cardiac, Thoracic, Vascular Sciences and Public Health, University of Padova, Padova, Italy; 5 Department of Biology, University of Padova, Padova, Italy; 6 Medical Oncology 3, Veneto Institute of Oncology IOV-IRCCS, Castelfranco Veneto, Italy; 7 Medical Oncology AULSS, 8 San Bortolo Hospital, Vicenza, Italy

**Keywords:** SCLC, Liquid biopsy, CfDNA, Chemoimmunotherapy, Atezolizumab

## Abstract

**Background:**

Atezolizumab (A) plus carboplatin-etoposide (CE) represents the new first-line treatment in extensive stage (ES)-Small Cell Lung Cancer (SCLC) patients. This study aims at identifying the association of baseline and dynamic changes of cfDNA, Tumor Fraction (TF) and variant allele frequency (VAF) of tumor-related mutations with median (m) overall (OS) and progression free survival (PFS) in SCLC patients treated with ACE.

**Materials and methods:**

This is a single-center prospective exploratory study including treatment-naive ES-SCLC patients eligible to first-line ACE. Liquid biopsies were longitudinally collected at baseline (T0), after cycle 1 (T1) and 2 (T2), at disease progression (T3). cfDNA Next Generation Sequencing (NGS) analysis was performed; genomic profiles and TF were inferred from shallow WGS (sWGS).

**Results:**

Thirty-two patients were included; mPFS and mOS were 5.19 and 7.96 months, respectively. Higher T0 cfDNA (HR 1.44, 95% CI 1.17–1.77, *p* = 0.0006) and VAF (HR 2.6, 95% CI 1.36–4.93, *p* = 0.0039) were associated with risk of death; higher T0 cfDNA (HR 1.29, 95% CI 1.08–1.54, *p* = 0.0049), TF (HR 1.97, 95% CI 1.02–3.82, *p* = 0.044) and VAF (HR 2.32, 95% CI 1.22–4.42, *p* = 0.01) were predictors of risk of PD. Among the dynamic changes in the biomarkers under investigation, the association of 10-unit increase of VAF T0-T1 and T0-T2 with OS (HR 1.38, 95% CI 1.01–1.88, *p* = 0.043; HR 1.56, 95% CI 1.21–2.16, *p* = 0.008) and PFS (HR 1.69, 95% CI 1.18–2.43, *p* = 0.004; HR 1.81, 95% CI 1.22–2.70, *p* = 0.003) was estimated.

**Conclusion:**

T0 and dynamic changes of cfDNA, TF and VAF may help physicians to stratify ES-SCLC patients receiving first-line ACE and to anticipate the clinical course of the disease.

**Supplementary Information:**

The online version contains supplementary material available at 10.1186/s13046-025-03434-3.

## Background

Small cell lung cancer (SCLC) is a high-grade neuroendocrine disease which occurs in approximately 15% of lung cancer patients [1]. At diagnosis, about 70% of SCLC patients present with extensive-stage (ES) disease [2]characterized by an aggressive phenotype and a dismal prognosis. Platinum-based chemotherapy has represented the standard of care for many years. Despite an initial high responsiveness to the treatment, the sensitivity is temporary, with a median progression-free survival (PFS) of 5–6 months and a median overall survival (OS) of 9–10 months [3]. Recently, immune checkpoint inhibitors combined with platinum-based chemotherapy emerged as the new first-line treatment in ES-SCLC patients [4–8]. In the IMpower133 trial, the addition of atezolizumab to platinum-based chemotherapy for 4 cycles followed by atezolizumab maintenance, showed to prolong the median overall survival (mOS) to 12.3 *versus* 10.3 months (hazard ratio [HR] 0.70; 95% confidence interval [CI] 0.54–0.91) with chemotherapy alone [5]. Similarly, the CASPIAN trial demonstrated the benefit of the addition of durvalumab to platinum-based chemotherapy, achieving a mOS of 12.9 *versus* 10.5 months (HR 0.71; 95% CI, 0.60–0.86) with chemotherapy alone [4].

Despite this promising data, the long-term benefit from the addition of immunotherapy to platinum-based chemotherapy is still an unmet medical need, with only 12% (95% CI, 7–17%) of patients alive at 5 years in the IMpower133/IMbrella A trial [9].

Furthermore, in some patients there is a lack of benefit from the addition of immunotherapy [5]. In this scenario, many efforts have been made by exploring novel potential biomarkers capable of predicting the outcome of patients and helping physicians in patients’ selection. However, finding new biomarkers in SCLC may be challenging due to the aggressive nature of the disease that often precludes the availability of adequate and sequential tissue specimens for in-depth studies [10].

Moreover, classical predictive factors of response to immunotherapy, such as programmed death-ligand 1 (PD-L1) and tumor mutational burden (MTB), do not appear to play a significant role in patients’ stratification [7, 11–16].

Conversely, the gene expression profiling (GEP) and the tumor microenvironment (TME) characterization may contribute to the identification of patients who could benefit from immunotherapy, as emerged from recent studies and explored by our group [17, 18].

With the aim of overcoming the issue of lacking tissue, in the last few years there has been an increasing development in liquid biopsy, now representing a reliable, accessible and non-invasive tool applicable in many settings. A relevant advantage of liquid biopsy is also its ability to catch tumor heterogeneity and its applicability in longitudinal studies. In this setting, serial blood samples could catch the plasticity and dynamic changes of SCLC, thus providing both qualitative and quantitative data on circulating tumor DNA [19]. To date, however, only few data are available on the predictive role of liquid biopsy in SCLC [20–22].

Based on these premises, we designed and conducted the CATS/ML43257 prospective study to identify circulating predictive biomarkers of response to chemoimmunotherapy in ES-SCLC patients, detected through longitudinal liquid biopsies, and potentially useful for patient stratification and predicting long-term benefit from immunotherapy.

## Methods

### Study design and eligibility criteria

**Fig. 1:**
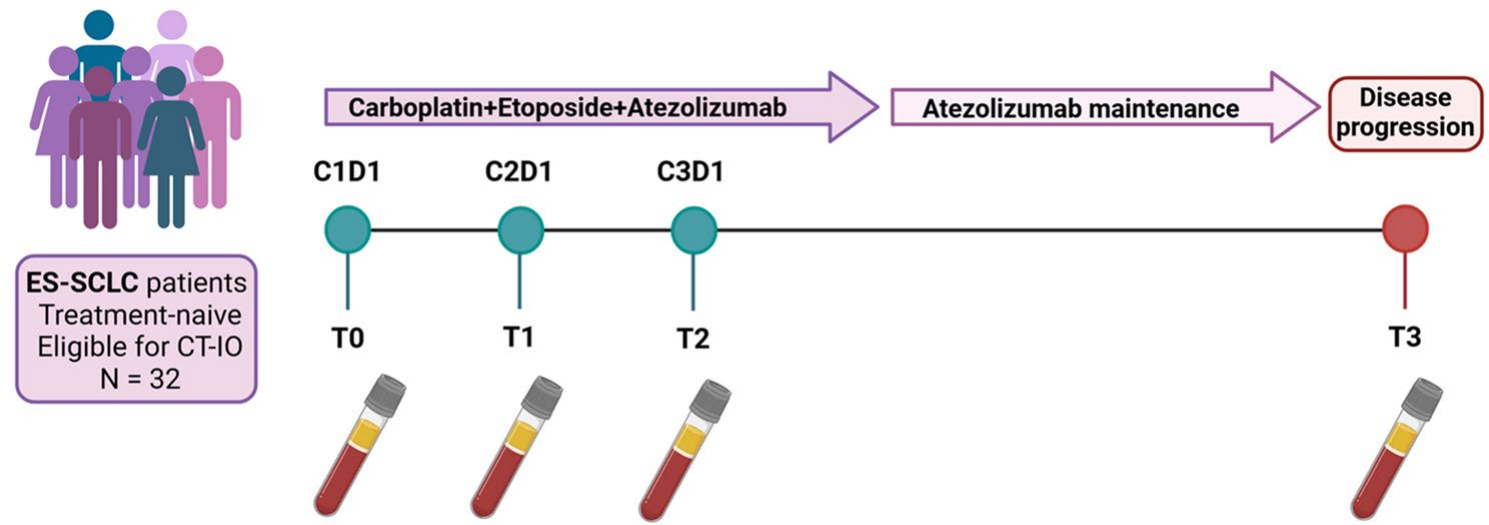
**Study design:** T0: baseline; T1 after first cycle; T2: after second cycle; T3 at the time of disease progression

This is a single-center exploratory observational translational study including treatment-naive patients affected by ES-SCLC eligible to first line treatment with atezolizumab plus carboplatin and etoposide (ACE). The study was conducted in accordance with Good Clinical Practice guidelines and the Declaration of Helsinki and approved by the Institutional Ethical Committee. All patients signed an informed consent form before enrollment. Patients showing symptomatic, actively progressing CNS metastases or leptomeningeal disease and those with contraindications to immune checkpoint inhibitors have been excluded. The study included 30 months of enrollment and at least 12 months of follow-up. Study design is depicted in Fig. 1.

Treatment was performed according to standard clinical practice and was based on induction phase with intravenous administration of carboplatin AUC 5 on day 1 every 21 days plus etoposide 100 mg/sm on days 1, 2, 3 every 21 days plus atezolizumab 1200 mg flat dose on day 1 every 21 days for a maximum of 4 planned cycles. Following the induction phase, each patient under disease control received atezolizumab maintenance until unacceptable toxicity, disease progression or loss of clinical benefit according to clinical practice.

Brain, Chest and Abdomen CT-scan for tumor assessment was performed at the baseline and every three cycles, according to clinical practice. Response rate was assessed according to Response Evaluation Criteria in Solid Tumors (RECIST) version 1.1. Safety was collected according to Common Toxicity Criteria for Adverse Events (CTCAE) version 5.0.

According to the study protocol, the primary endpoint of the study was the association between baseline cfDNA and its variations at different timepoints and progression free survival (PFS). The secondary endpoints were: (i) the association between baseline cfDNA and its dynamic variation and overall survival (OS); (ii) the association between baseline TF and VAF and their dynamic variation with PFS and OS; (iii) the characterization of tumor-related mutations in the study population through liquid biopsy, both at baseline and at the time of disease progression.

### Plasma collection and cfDNA extraction

Liquid biopsies were collected longitudinally at key timepoints: T0 (baseline evaluation, corresponding to the first day of the first treatment cycle), T1 (after the first cycle of therapy, corresponding to the first day of the second treatment cycle), T2 (after the second cycle of therapy, corresponding to the first day of the third treatment cycle), and T3 (at disease progression). For each collection, approximately 20 mL of peripheral blood was drawn from each patient into two Cell-Free DNA BCT tubes (Streck Corporate, La Vista, NE, USA) and processed within 24 to 72 h. Blood samples underwent a two-step centrifugation process: first at 2000×g for 10 min at 4 °C, followed by 20,000 g for 10 min. Plasma aliquots were stored at − 80 °C until analysis, as detailed previously [23].

Cell-free DNA (cfDNA) was extracted from 2 to 5 mL of plasma using the AVENIO cfDNA Isolation Kit (Roche Sequencing Solutions, Pleasanton, CA) according to the manufacturer’s protocol. The extracted cfDNA was then quantified fluorometrically using the Qubit dsDNA HS Assay Kit (Thermo Fisher Scientific, Waltham, MA, USA).

### cfDNA next generation sequencing analysis

Next Generation Sequencing (NGS) libraries were prepared from 10 to 50 ng of cfDNA in a total volume of 50 µL using the AVENIO ctDNA Expanded kit v2 (77 genes; Roche Diagnostics Spa) according to the manufacturer’s instructions as previously described [23]. Individual enriched libraries were quantified using the QuBit dsDNA HS Assay kit (Thermo Fisher Scientific), and their profile was assessed using the Agilent High Sensitivity kit on the Agilent 2100 Bioanalyzer. Each sequencing run comprised a pooled set of 8 samples, processed on an Illumina NextSeq 500/550 platform using the 300-cycle NextSeq High Output kit in paired-end mode (2 × 150 cycles). The sequencing-generated FastQ files were analyzed using the AVENIO ctDNA analysis software (Roche Diagnostics) with default parameter settings for the Expanded Panel V2. Following the manufacturer’s guidelines, the percentage of reads aligned to the human reference genome (hg38) within the targeted region (unique depth) should exceed 40%. Additionally, the median unique depth across bases in the targeted region should be at least 2500×. Variant allele frequency (VAF) was calculated as the ratio of mutated reads to the total sequence reads at a given genomic position.

All identified variants were manually reviewed. Synonymous, intronic variants and those found in population databases such as ExAC and dbSNP as polymorphisms were excluded. Coding protein and splicing variants were evaluated for pathogenicity using Varsome Premium (Saphetor SA, Lausanne, Switzerland). Only variants annotated as pathogenic, likely pathogenic or with uncertain/unknown significance (VUS) were considered as trackable mutations in plasma samples for longitudinal analyses.

### Shallow whole genome sequencing (sWGS) analysis

Whole genome libraries were prepared using 10 to 50 ng of cfDNA with the KAPA HyperPrep kit (Roche), following the manufacturer’s protocol, and sequenced on the Illumina NextSeq 500/550 platform (Illumina Inc, San Diego, CA, USA) using 75 bp single-end reads.

Genomic profiles and tumor fraction (TF) were inferred using both the SAMURAI and the ichorCNA algorithms, achieving a sensitivity of 0.95 and a specificity of 0.91 for tumor detection, with a TF cutoff of 0.03 (Limit of Detection, LOD = 3%) [24, 25].

### Statistical analysis

Continuous variables were summarized as medians and interquartile ranges (IQRs), while categorical variables were presented as frequencies and percentages.

Survival curves for overall survival (OS) and progression-free survival (PFS) were estimated using the Kaplan–Meier method, and median survival times were reported along with the corresponding 95% confidence intervals (CIs). Time-to-event was defined as the interval from the date of first treatment to the occurrence of death (OS and PFS) and/or disease progression (PFS), whichever occurs first. The median follow-up time was estimated using the reverse Kaplan-Meier method. The Mantel-Cox test was used to compare survival distributions between groups of patients with and without TF clearance at T2.

To assess the univariable association between baseline clinical variables, biomarkers, and survival outcomes, Cox proportional hazards (PH) models were fitted, and hazard ratios (HRs) with 95% CIs were reported. For continuous variables, HRs were computed per IQR increase to facilitate interpretation. The proportional hazards assumption was assessed using Schoenfeld residuals and graphical methods.

A dynamic analysis was conducted by modeling the change (deltas) in biomarkers levels over time, adjusting for baseline biomarkers levels. The deltas considered were the differences in biomarkers levels between T1 and T0, as well as between T2 and T0. Initially, restricted cubic splines were used to flexibly model the relationship between biomarker changes and hazards, accounting for potential non-linear effects. However, as a consistent linear relationship was observed—precluding the identification of meaningful cut-off values for the outcomes prediction—a simplified Cox PH model was subsequently used, estimating the HR with 95% CIs per 10-unit increase in biomarkers deltas (Supplementary Fig. 1).

As an exploratory analysis, a random forest algorithm was implemented to assess the relative importance of clinical and biomarker variables. Variable importance was evaluated using the permutation importance measure (VIMP), without further model selection or inference.

The required number of events was estimated considering cfDNA as a continuous covariate in a univariable Cox model, with PFS as the primary endpoint [26]. Assuming a hazard ratio (HR) of 1.5 for a 50-unit difference in cfDNA, corresponding to the interquartile range (Q1-Q3), given a standard deviation of 70, an expected event rate of 80%, 80% power, and a two-sided α = 0.05, the required number of events is approximately 24. This translates to a total sample size of ~ 30 participants (N = E/p).

All statistical analyses were performed using R version 4.4.2. A two-sided p-value < 0.05 was considered statistically significant.

## Results

### Patients’ characteristics

Overall, 33 patients were included in the CATS/ML43257 study between October 2021 and March 2024.

One patient was finally excluded from data analyses because of rapid clinical worsening and early start of best supportive care, without receiving chemo-immunotherapy. Clinical features of the 32 eligible patients are summarized in Table 1. In summary, patients were mostly males (*N* = 19, 59.4%), with an ECOG PS of 0 or 1 (*N* = 28, 87.5%) and a median age of 65 years. All patients reported a previous/current smoking history, with a median of 40 pack/years; 22 (68.8%) patients had less than three metastatic sites, and 7 (21.9%) had treated and/or clinically stable brain metastases at the diagnosis. Liver metastases were present at baseline in 6 (18.8%) patients and bone metastasis in 13 (40.6%) cases.

**Table 1:** Patients’ characteristics

	Overall (*N* = 32)
**Gender**	
Female	13 (40.6%)
Male	19 (59.4%)
**Smoking status**	
Current Smokers	20 (62.5%)
Former Smokers	12 (37.5%)
**Charlson Comorbidity Index**	
≤ 7	6 (18.8%)
> 7	26 (81.2%)
**Performance status**	
0	11 (34.4%)
1	17 (53.1%)
2	4 (12.5%)
**Age at diagnosis (years)**	
Median [IQR]	65 [57, 73]
**Stage at diagnosis**	
Limited	4 (12.5%)
Extended	28 (87.5%)
**Number of metastasis**	
< 3	22 (68.8%)
≥ 3	10 (31.2%)
**Extrathoracic metastasis**	
Absent	8 (25.0%)
Present	24 (75.0%)
**Liver Metastasis**	
Absent	26 (81.2%)
Present	6 (18.8%)
**Bone metastasis**	
Absent	19 (59.4%)
Present	13 (40.6%)
**Central nervous system metastasis**	
Absent	25 (78.1%)
Present	7 (21.9%)
**Molecular profile**	
NEUROD1+	2 (6.0%)
ASCL1+	3 (10.0%)
TRIPLE NEGATIVE	10 (31.0%)
Unknown	17 (53.0%)

Gene expression profiling on tumor tissue was previously performed in 15 (47%) patients: ten harbored an inflamed (triple negative, I), three an ASCL1+ (A) and two a neuroendocrine (NEUROD1+, N) molecular profile, as previously reported [17].

Median number of induction cycles were 4 (1–6), while patients received a median of 3 (0–43) cycles of maintenance. With a median follow-up of 20 months (95% CI 19.0 - not reached), the intention-to-treat (ITT) population showed an objective response rate of 75% and a disease control rate of 84.4%. The median progression-free survival (PFS) was 5.19 months (95% CI 4.74–6.55), and the median overall survival (OS) 7.96 months (95% CI 6.91–14.64) (Supplementary Fig. 2). Among the categorial clinical features, gender (males HR 2.44, 95% CI 1.01–5.88, *p* = 0.047) and the number of metastatic sites (ms) (*≥* 3 h 2.90, 95% CI 1.18–7.09, *p* = 0.020) significantly associated with OS, while the number of ms was also associated with PFS (*≥* 3 h 3.44, 95% CI 1.44–8.23, *p* = 0.005) (Table 2).

**Table 2:** Association of categorial clinical features with PFS and OS

Cox Proportional Hazard Model							
		Progression Free Survival			Overall Survival		
Sex		HR	95% CI	*p*-value	HR	95% CI	*p*-value
	Female	-	-		-	-	
	Male	1.26	0.58, 2.73	0.6	2.44	1.01, 5.88	**0.047**
**Smoking Status**							
	Former smoker	-	-		-	-	
	Current smoker	1.02	0.47, 2.20	> 0.9	1.27	0.56, 2.90	0.6
**Charlson Comorbidity Index**							
	≤ 7	-	-		-	-	
	> 7	1.26	0.48, 3.35	0.6	1.45	0.50, 4.26	0.5
**Performance Status**							
	≤ 1	-	-		-	-	
	> 1	1.41	0.48, 4.19	0.5	1.38	0.39, 4.92	0.6
**Number of metastasis**							
	< 3	-	-		-	-	
	≥ 3	3.44	1.44, 8.23	**0.005**	2.90	1.18, 7.09	**0.020**
**Extra-thoracic metastasis**							
	No	-	-		-	-	
	Yes	1.37	0.58, 3.27	0.5	0.93	0.38, 2.29	0.9
**Liver metastasis**							
	No	-	-		-	-	
	Yes	2.11	0.84, 5.33	0.11	1.91	0.70, 5.22	0.2
**Bone metastasis**							
	No	-	-		-	-	
	Yes	1.65	0.77, 3.50	0.2	1.07	0.46, 2.44	0.9
**Brain metastasis**							
	No	-	-		-	-	
	Yes	1.20	0.48, 2.98	0.7	1.28	0.48, 3.45	0.6

No significant difference in terms of baseline cfDNA (*p* = 0.12), TF (*p* = 0.18) and VAF (*p* = 0.14) was observed between the inflamed and the neuroendocrine *plus* ASCL1 + phenotype (data not shown), probably because of the small sample size.

### cfDNA analysis

We collected 32 samples at T0 and T1, 31 samples at T2 (reduction due to patient’s death), and 21 samples at T3. Specifically, at the data cut-off (31st October 2024), 6 patients had died before T3 sampling, and 5 patients had not yet progressed. cfDNA was obtained in all 116 plasma samples collected across the timepoints. At diagnosis (T0), the median cfDNA level was 24.9 ng/mL of plasma, respectively, ranging from 3.7 to 704.3 ng/mL.

**Fig. 2:**
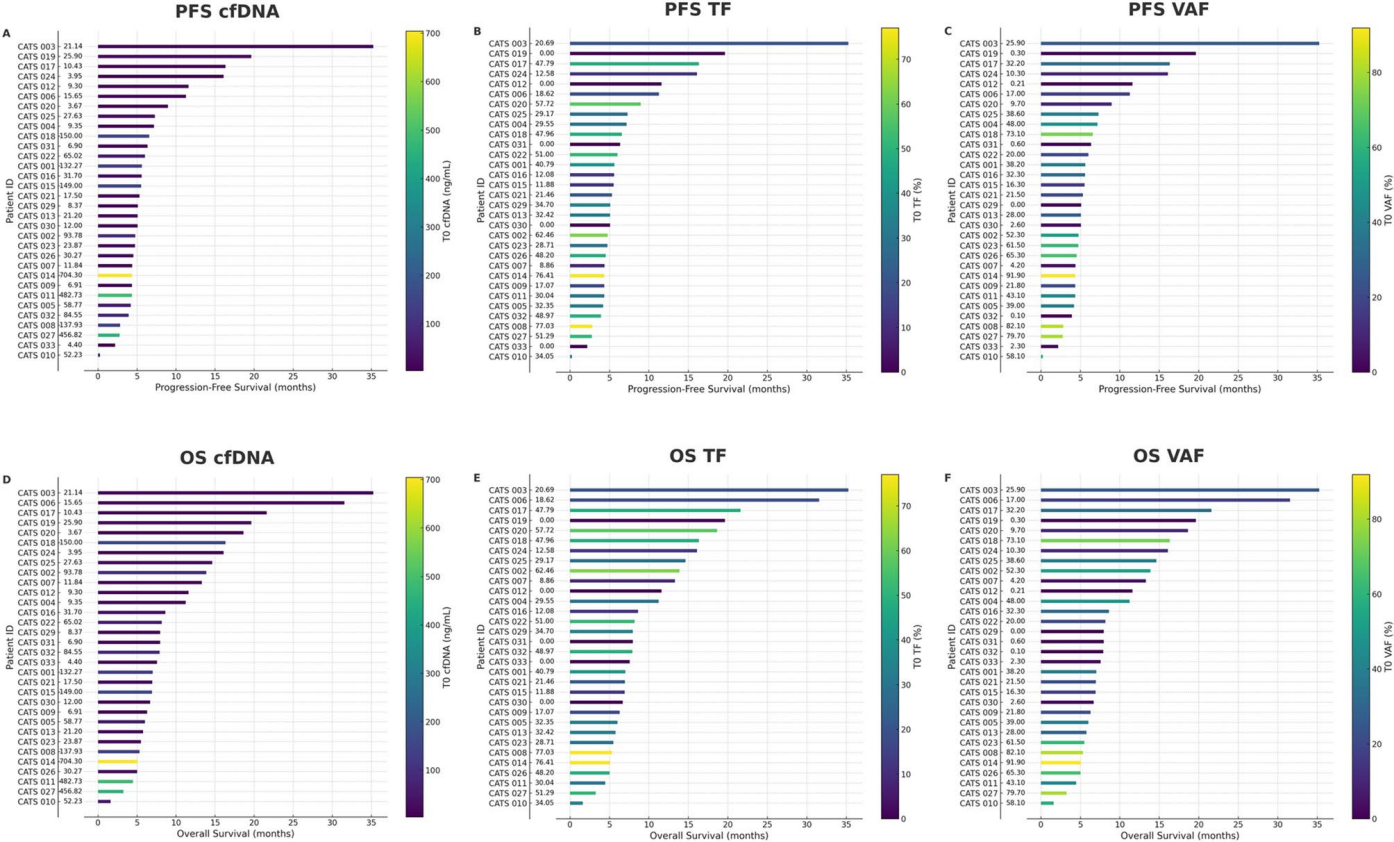
Swimmer plot showing association between baseline values of cfDNA, TF and VAF with PFS (**A**–**C**) and OS (**D**–**F**). The values reported on the Y axis between the patients’ ID and the plots are the T0 values of cfDNA (**A**, **D**), TF (**B**, **E**) and VAF (**C**, **F**)

Baseline cfDNA was considered as a continuous variable ranging from 10.16 ng/mL (Q1) to 86.86 ng/mL (Q3) in association with PFS and OS. Higher levels of baseline cfDNA were significantly associated with both disease progression (HR 1.29, 95% CI 1.08–1.54, *p* = 0.0049) and higher risk of death (HR 1.44, 95% CI 1.17–1.77, *p* = 0.0006) (Fig. 2).

**Fig. 3:**
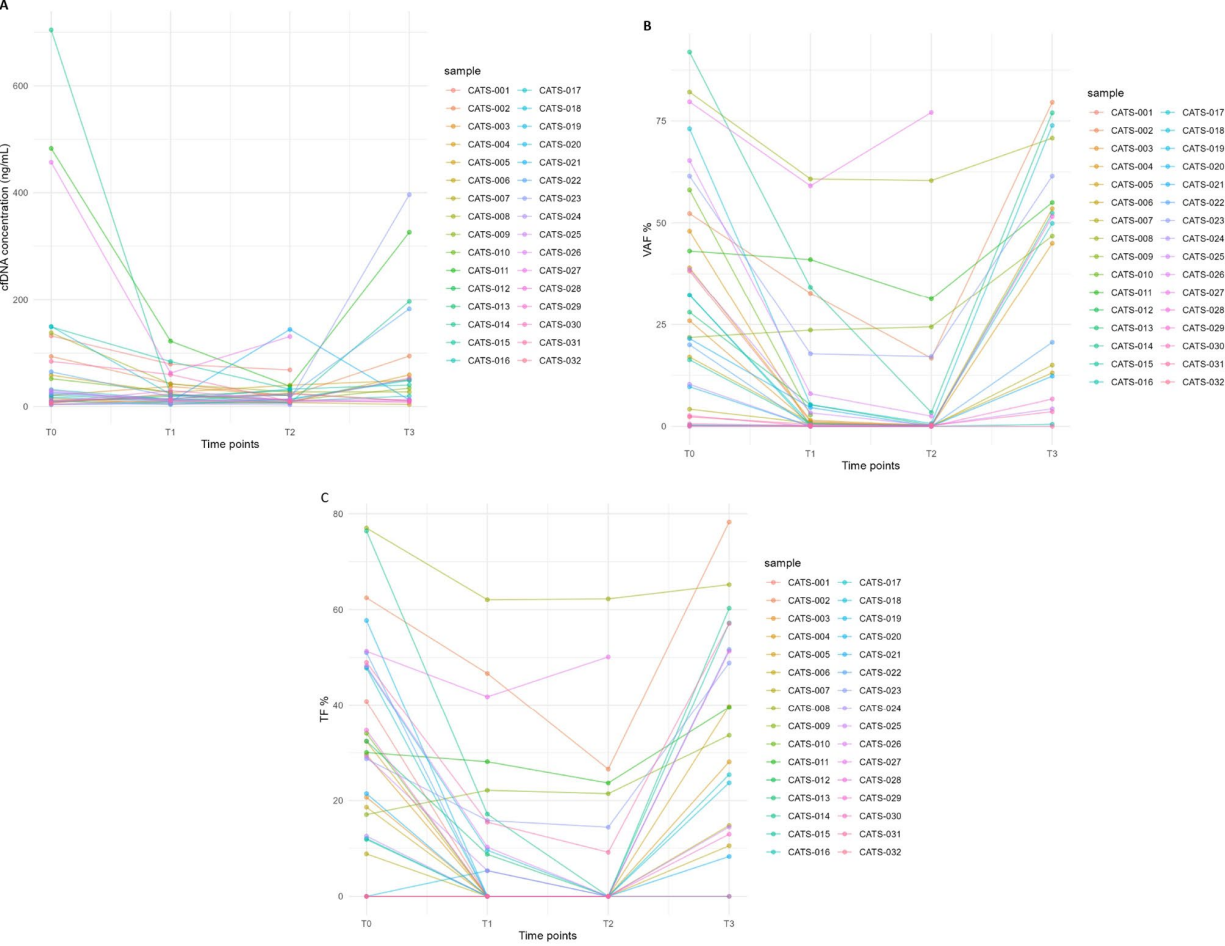
Spaghetti plot showing dynamic changes of cfDNA (**A**), VAF (**B**) and TF (**C**) along different timepoints in each patient

Furthermore, a significant reduction in cfDNA levels was observed between T0 and T1 (median 24.9 ng/mL, Q1-Q3: 10.2–86.9 vs. median 19.1 ng/mL, Q1-Q3: 11.3–31.9; *p* = 0.03) and between T0 and T2 (median 24.9 ng/mL, Q1-Q3: 10.2–86.9 vs. median 14.2 ng/mL, Q1-Q3: 9.9–28.8; *p* = 0.03). At the time of disease progression (T3), cfDNA levels showed a significant increase relative to T2 (median 14.2 ng/mL, Q1-Q3: 9.9–28.8 vs. median 40.5 ng/mL, Q1-Q3: 12.5–59.3; *p* = 0.01), sometimes reaching values like those observed at baseline (Fig. 3a). The increase of cfDNA levels at different timepoints was not significantly associated with PFS, whereas a higher risk of death was estimated in presence of a dynamic 10-unit increase of the change in cfDNA between T0-T2 (HR 1.17, 95% CI 1.04–1.32, *p* = 0.009).

### Characterization of tumor-related mutations

**Fig. 4:**
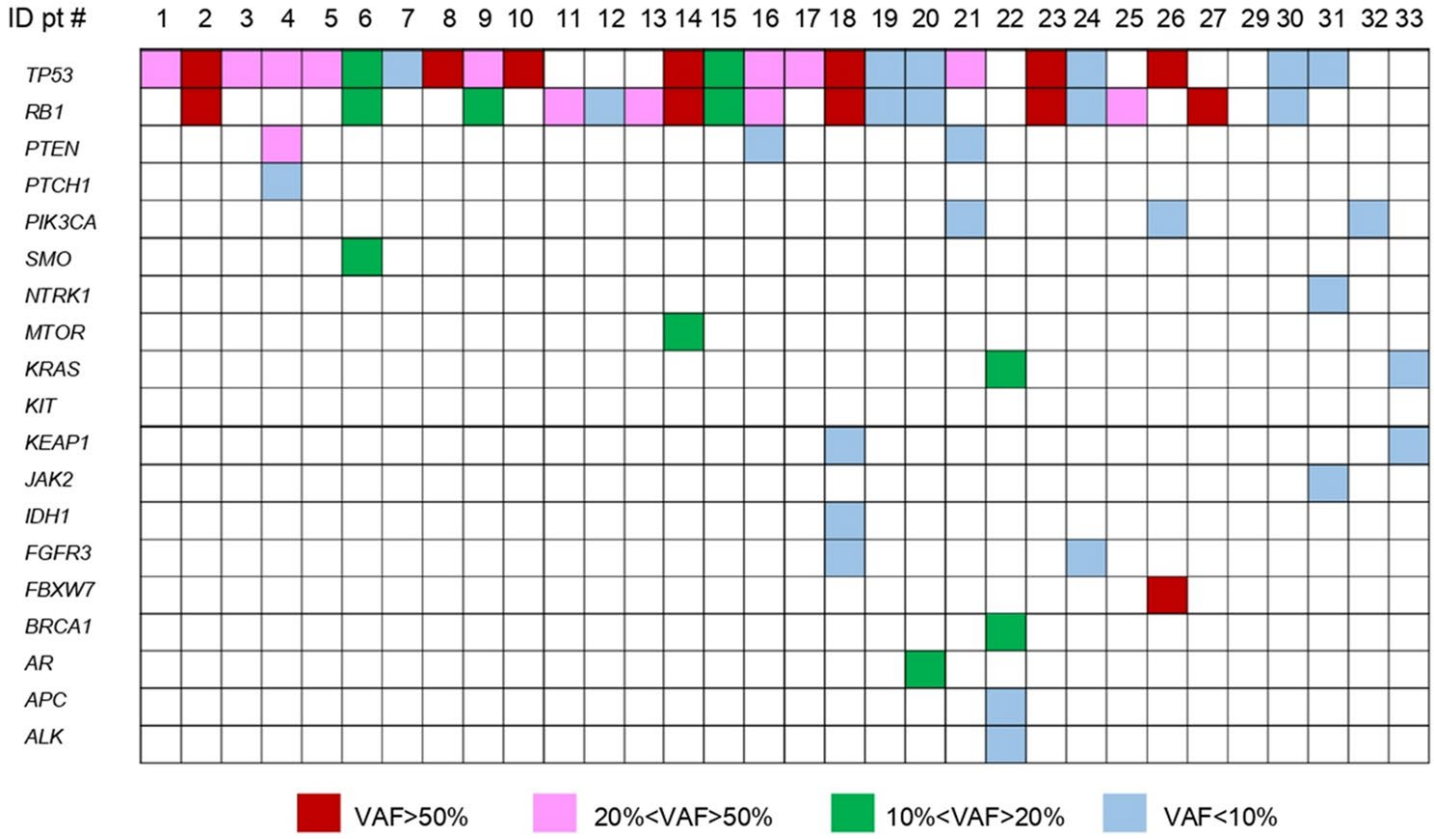
Heatmap showing tumor-related mutations and specific VAF detected at T0

Tumor-related mutations were identified in 31 out of 32 patients at T0 (96.8%). Among these, 19 patients (61.2%) exhibited two or more pathogenic or likely pathogenic genetic alterations. *TP53* and *RB1* were the most frequently altered genes. Specifically, 23 (74%) patients exhibited *TP53* alterations, and 17 (54.8%) had *RB1* mutations. *TP53* mutations predominantly involved missense alterations in the DNA-binding domain, while *RB1* mutations were primarily nonsense mutations. Other frequently altered genes included *PTEN* (9.3%), *PIK3CA* (9.3%) and *KRAS* (6.2%). A list of mutated genes with specific variant allele fraction (VAF) is shown in Fig. 4. The gene with the highest VAF was used to evaluate clearance (a 99–100% reduction in VAF) during treatment. VAF clearance was observed in 12 (38.7%) patients between T0 and T1 and in 21(67.7%) patients between T0 and T2. Conversely, at the time of disease progression (T3), an increase of the VAF of tumor-related mutations, whose values returned similar to those at the baseline, was observed in 16 patients (50%). Additionally, new pathogenic mutations emerged in 8 (25%) patients, each with a VAF < 10% (Table 3). Moreover, no significant differences in terms of median PFS and OS were observed between patients who, at progression, exhibited new pathogenic variants compared to those at diagnosis (data not shown).

**Table 3:** Pathogenic mutations detected at progression with their variant allele frequency (VAF). Variants shown in bold emerged at progression, whereas those in regular black font were already identifiable at the time of diagnosis

Patient	Pathogenic mutation	VAF (%)
CATS 1	*N.A.*	
CATS 2	*RB1* c.751 C > T; *TP53* c.785G > T	78.3; 79.6
CATS 3	*N.A.*	
CATS 4	***ALK*** **c**.**1414** + **1G** > **A**; *PTCH1* c.2560 + 1G > A; *PTEN* c.375 A > T; *TP53* c.375 G > T	**0**.**27**; 0.9; 42.7; 45.0
CATS 5	***JAK*** **c**.**1744** **C** > **T**; *TP53* c.578 A > G	**0**.**36**; 53.5
CATS 6	*RB1* c.1498 + 2T > G; *SMO* c.1265 G > T; *TP53* c.329 G > T	11.8; 9.4; 13.1
CATS 7	*TP53* c.427 G > C; *TP53* c.848 G > C	0.25; 15
CATS 8	***ERBB2*** **c**.**932 G** >**A**; *TP53* c.542 G > C	**0**.**14**; 70.8
CATS 9	*RB1* c.717T >A; *TP53* c.797 G >T	42; 46.8
CATS 10	*N.A.*	
CATS 11	***TP53*** **c**.**707** **A** >**T**; *RB1* c.1694 C >G	**0**.**12**; 55
CATS 12	*N.A.*	
CATS 13	*N.A.*	
CATS 14	*MTOR* c.5930 C > G; *RB1* c.1422–2 A >T; *TP53* c.476 C > T	34.3; 77; 70.5
CATS 15	***RB1*c**.**1422**–**2** **AC** >**T**; *RB1* c.1333 C > T; *TP53* c.482 C > A	**0**.**7**; 52.4; 51.1
CATS 16	*PTEN* c.319G > T; *RB1* c.1215 + 1G > T; *TP53* c.737 T > G	0.2; 0.54; 0.5
CATS 17	*TP53* c.976G > T	49.9
CATS 18	***SMAD4*c**.**1487 G** >**T**; *KIT* c.1255 G > C; *RB1* c.1960 + 1G >A; *TP53* c.743 G > T	**0**.**4**; 9.6; 71.8; 73.9
CATS 19	*N.A.*	
CATS 20	*N.A.*	
CATS 21	***APC*c**.**4648G** >**T**; *TP53* c.586 C >T;	**8**.**6**; 12.3
CATS 22	*BRCA1* c.4148 C >A; *FGFR1* c.1591 C >G; *KRAS* c.29G > C; *ALK* c.2725 G >A	20.6; 0.2; 20.2; 0.8
CATS 23	*RB1 c.2212-1G> A;* *TP53 c.314G >T*	60.6; 61.5
CATS 24	*N.A.*	
CATS 25	***GNAQ*c**.**196G** >**T**; *RB1* c.2027T > G	**0**.**2**; 4.3
CATS 26	*N.A.*	
CATS 27	*N.A.*	
CATS 29	*No variants identified*	
CATS 30	*RB1* c.2158 A > T; *TP53* c.701 A > G	6.7; 6.6
CATS 31	*TP53* c.578 A > G	3.6
CATS 32	*No variants identified*	
CATS 33	*N.A.*	

N. A: Not Available

Ranging from the first quartile (8.33%) to the third quartile (49.08%), higher baseline VAF was significantly associated with higher risk of disease progression (HR 2.32, 95% CI 1.22–4.42, *p* = 0.01) and death (HR 2.59, 95% CI 1.36–4.93, *p* = 0.0039) (Fig. 2).

The dynamic trend of VAF was observed through longitudinal analysis; the median VAF at T0, T1, T2, and T3 was 27.0% (Q1–Q3: 8.3–49.1), 0.9% (Q1–Q3: 0–6.0), 0.2% (Q1–Q3: 0–1.6), and 46.8% (Q1–Q3: 12.3–55.0), respectively. A statistically significant VAF clearance was observed between T0 and T1 (*p* = 0.0002) and between T0 and T2 (*p* = 0.0001). Conversely, a statistically significant increase in VAF was detected between T1 and T3 (*p* = 0.0004) and between T2 and T3 (*p* = 0.0002) (Fig. 3b).

A higher risk of disease progression was estimated in presence of a dynamic 10-unit increase of change in VAF between T0-T1 (HR 1.69, 95% CI 1.18–2.43, *p* = 0.004) and T0-T2 (HR 1.81, 95% CI 1.22–2.70, *p* = 0.003), and a higher risk of death with a 10-unit increase of change in VAF between T0-T1 (HR 1.38, 95% CI 1.01–1.88, *p* = 0.043) and T0-T2 (HR 1.56, 95% CI 1.21–2.16, *p* = 0.008) was observed.

### Characterization of TF and genomic profiles by shallow whole-genome sequencing (sWGS)

sWGS revealed altered genomic profiles with a positive tumor fraction (TF ≥ 3%) in most of the patients both at baseline (T0) and disease progression (T3): among the samples collected, 27 out of 32 (84.4%) and 19 out of 21 samples (90.5%) exhibited a positive TF at T0 and T3 respectively. At interim timepoints T1 and T2, altered genomic profiles with positive TF were observed for 13 out of 32 samples (40.6%) and in 7 out of 31 samples (22.6%) at T1 and T2. Detailed TF values for each timepoint are provided in Supplementary Table.

The median TF at T0 was 29.8% (Q1-Q3: 12.5–48%); the median TF dropped to 0.0% (Q1-Q3: 0.0-11.6%) at T1 and at T2 (Q1-Q3: 0.0–0.0%), and rose again to 33.6 (Q1-Q3:14.5–51.6%) (Fig. 3c).

The only cases with negative TF at T3 were CATS 16 and CATS 31.

Ranging from the first (12.46%) to the third (48.02%) quartile, higher baseline TF was a predictor of higher risk of disease progression (HR 1.97, 95% CI 1.02–3.82, *p* = 0.044) (Fig. 2).

Among the 27 patients with positive TF at T0, two distinct patterns emerged: 20 patients (20 out of 27, 74%), hereunder referred to as first group, showed normalization of genomic profiles accompanied by complete clearance at T2 (100% TF reduction). In contrast, seven patients (7 out of 27, 26%), hereunder referred to as second group, retained altered genomic profiles and showed incomplete TF clearance, with statistically significant TF reductions from 3.3 to 81.2% at T2 or, in one case (CATS 9), even a TF increase (Fig. 3; Supplementary Fig. 3).

In the first group, the median TF at T0 was 32.39% (Q1-Q3: 20.2–47.8%); at T1 it set down significantly to 0.00% (Q1-Q3: 0.00–1.3%), as well as at T2 (median 0.00); while at progression (T3) the median TF rose again to 25.41% (Q1-Q3: 14.5–51.3%). However, in this group TF levels increased again at T3 for all but two patients (CATS 16 and CATS 31).

In the second group, the median TF at T0 was 48.97% (Q1-Q3: 29.4–56.9%); at T1 it decreased slightly in six patients (CATS 2, CATS 8, CATS 11, CATS 23, CATS 27, CATS 32), with reductions ranging from 6.42 to 68.37%, while it increased in one patient (CATS 9) by 29.76%. The median TF at T1 was 28.11% (Q1-Q3: 19-44.2%); at T2 it decreased in five patients (CATS 2, CATS 9, CATS 11, CATS 23, CATS32) by 3.21–43.05% but increased in the remaining two patients by 0.29–20%, with a median TF of 23.66% (Q1-Q3: 17.9–38.3%). By T3, all patients of this group exhibited rising TF, with a median of 52.96% (Q1-Q3: 41.8–63.2%).

The 20 patients in the first group, showing complete TF clearance at T2, had notably longer PFS (*p* = 0.0009) compared to the 7 patients in the second group with incomplete TF clearance.

Finally, a third group is composed of five patients (CATS 12, CATS 19, CATS 30, CATS 31, CATS 33), who displayed a TF of 0.00% and normal genomic profiles at T0. Apart from CATS 19, who displayed a TF of 5.36% at T1, all these patients consistently showed negative TF values at both T1 and T2. In this small group of patients, T3 samples were available only for CATS 30 and CATS 31, with TF values of 12.97% and 0.00%, respectively.

A higher risk of disease progression in presence of a dynamic 10-unit increase in change of TF between T0-T1 (HR 1.49, 95% CI 1.1–2.01, *p* = 0.01) and T0-T2 (HR 1.86, 95% CI 1.25–2.75, *p* = 0.002) was observed, as well as a higher risk of death in presence a 10-unit increase of TF between T0-T2 (HR 1.56, 95% CI 1.09–2.21, *p* = 0.014), was estimated.

**Fig. 5:**
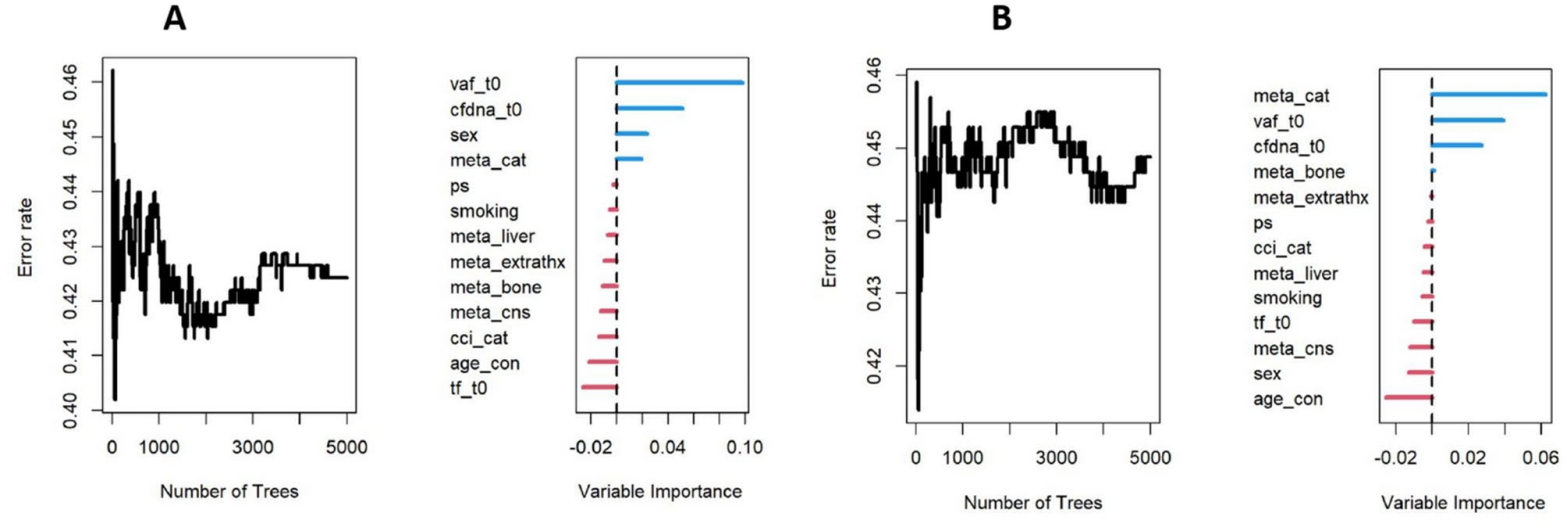
Random forest of covariates impact on OS (**A**) and PFS (**B**) and their relevance

Lastly, for exploratory purposes, we implemented a random forest algorithm that included all the covariates. This analysis identified baseline cfDNA, VAF and the number of metastases as important predictors of both OS and PFS (Fig. 5).

## Discussion

Small cell lung cancer is biologically characterized by high tumor heterogeneity and plasticity, thus explaining the clinical aggressiveness and dismal outcome from available systemic treatments [27]. Even though a new standard of care has been introduced as first line treatment, long-term benefit from chemoimmunotherapy is reserved to a small patients’ subgroup, which is tough to identify from a clinical perspective [28]. The modest progression free and overall survival shown by our real-world cohort of patients confirmed these data and are in line with available literature from clinical trials.

Recently, transcriptomic profiling on tumor samples of SCLC has defined 4 different subtypes, characterized by positivity to ASCL1 (A), NEUROD1 (N), POU2F3 (P) transcription factor signatures or by the lack or low expression of these three signatures and associated with an inflammatory gene expression signature (SCLC-I) [18]. Although available data show a differential benefit from chemoimmunotherapy according to the different subgroups [28, 29]this classification is not yet currently used in clinical practice.

In this clinical setting, the search for tissue predictive biomarkers is difficult to pursue because of the paucity of tumor tissue and the dynamic evolution of the disease [10]whereas liquid biopsy approaches are valuable also considering that SCLC is an highly tumor DNA shedding cancer, as demonstrated by the higher concentration of ctDNA compared with plasma samples of Non-Small Cell Lung Cancer and other tumor types [22, 30, 31]. Tumor DNA shedding is determined by cancer type and other biological and clinical features, such as the number and location of the metastatic site [32, 33].

In line with previous findings, we detected a median cfDNA concentration of 24.9 ng/mL of plasma (range 3.7-704.3 ng/mL) and a median TF of 29.8% (Q1-Q3: 12.5–48%) in our case series, whereas in NSCLC only 20% of cases have TF exceeding 10%^30^. These data reinforce the potential usefulness of liquid biopsy to detect and monitor cfDNA during systemic SCLC treatment. The prognostic role of cfDNA in cancer has been reported in many studies [34]and our data confirm this in SCLC unveiling an association of increasing cfDNA concentration with the risk of death ([*p* = 0.0006], with an estimated higher risk of death in presence of a dynamic 10-unit increase of the change in cfDNA between T0-T2 [*p* = 0.009]), probably because of a direct correlation with the number of metastases. Additionally, our data showed an association of cfDNA levels with the risk of disease progression to first line chemoimmunotherapy.

The attitude of SCLC to release ctDNA enabled the successful measurement of the TF by sWGS, a well-established method in the field to quantify the proportion of cancer derived cfDNA in plasma samples [35].

In our study, higher baseline TF and a 10-unit increase in TF levels between T0 and T1 or T0 and T2 were associated with an elevated risk of disease progression (*p* = 0.044; *p* = 0.01; *p* = 0.002), while a 10-unit TF increase between T0 and T2 correlated with a higher risk of death (*p* = 0.014). Notably, TF clearance at T2 in our cohort was linked to improved PFS (*p* = 0.0009). These findings are consistent with previous research on different cancer types, suggesting that baseline TF and its dynamic changes can serve as prognostic and predictive biomarkers [36, 37].

Notably, a small subgroup of 5 patients showed absence of TF in the baseline plasma samples, along with a lower cfDNA concentration compared to the rest of the cohort (median 9.3 ng/mL of plasma versus 28.95 ng/mL of plasma). In these patients, however, somatic point mutations were detected by the Avenio NGS test, with a VAF ranging from 0.21 to 2.6%. These VAF values fall below the limit of detection of the algorithms used for TF calculation, which requires a TF of at least 3%^24,25^, confirming the low level of ctDNA in these plasma samples. Considering the undetectable TF, the low plasmatic cfDNA yield and the low VAF of the driver mutations observed in this cohort, we considered these patients to be low ctDNA shedders.

In this respect, a previous large study by Husain et al. reported a median TF of 17.7% in liquid biopsies of SCLC patients. Notably, out of 115 patients, TF was undetectable for 12% of the samples analyzed. These results align with our findings, where TF was undetectable in 5 out of 32 samples (15.6%). Low or undetectable TF has been attributed to insufficient ctDNA shedding or limitations in assay sensitivity [30, 38].

Notably, all these 5 SCLC patients had less than 3 metastatic sites, bone metastases were present in 2 (40%) patients, brain metastasis in 1 (20%) patient and no one had liver metastasis at baseline. It is possible to hypothesize that TF-negative SCLC may exhibit a distinct biological tumor behavior, characterized by a lower propensity for metastasis. However, the lack of available tissue prevented further investigation of this hypothesis.

In addition to the prognostic value of static values of these biomarkers, the clinical value of dynamic monitoring of cfDNA is even more attractive from a clinical perspective, because the change in concentration over time may be useful especially in the first cycles of treatment, because it may help physicians to anticipate the radiological detection of disease progression. The analysis of the relationship between changes in cfDNA levels across different time points and survival outcomes, conducted using restricted cubic splines, revealed an approximately linear association with the log hazard ratio. This finding supported our decision to treat cfDNA clearance as a continuous variable in survival analyses, rather than defining an arbitrary cut-off for patient stratification.

As in other settings [39]our data showed a significant reduction in cfDNA levels between T0 and T1 (median 24.9 ng/mL vs. 19.1 ng/mL) and between T0 and T2 (median 24.9 ng/mL vs. 14.2 ng/mL), while at T3 cfDNA levels showed a significant increase compared to T2 (median 14.2 ng/mL vs. 40.5 ng/mL). These data acquire even more clinical relevance when we consider the rapid disease progression and clinical worsening of SCLC patients; in our series, 11 patients (34%) were not eligible to the blood collection at the T3 time point, because of rapid PS deterioration and death.

In our case series, we identify two patients without TF detected in the plasma samples collected at the time of disease progression. One patient was classified as a low shedder patient, with undetectable TF at all timepoints; while the other showed initially a positive TF at T0 (12.8%), that dropped to 0 at subsequent timepoints.

Both patients had less than 3 metastatic sites at diagnosis with respectively lymph nodes and pleural involvement, without bone, brain or liver metastasis: at T3 they both experienced systemic disease progression with brain metastasis in one case and lymph nodes disease in the other case.

Our study reports frequent *TP53* and *RB1* gene mutations at the baseline, thus allowing their monitoring along treatment cycles; significant association of both baseline values and dynamic deltas were shown with OS and PFS. Previous studies from our group [40, 41] and others [42, 43] underlined the clinical applicability of VAF detection; the identification of a small number of specific mutations and their adoption as sentinel of clinical cancer course could hopefully make this tool accessible and cost-saving compared with repeated wide-genome sequencing [44]. Interestingly, *PTEN* mutations have been detected in three patients, and only in one patient with a VAF of 20–50%. Interestingly, this patient developed brain metastases at the time of disease progression, in line with findings of a previous study [45]suggesting that specific pathway alterations might correlate with progression patterns.

Finally, an additional insight offered by the dynamic monitoring of gene mutations in liquid biopsies of SCLC patients is the identification of acquired resistance mutations at the time of disease progression which could be hypothesis-generating for further studies on resistance mechanisms and signature after immune checkpoint inhibitors. Acquired mutation of the *APC* gene has been observed at high VAF in one patient, who experienced a clinical course of aggressiveness and early death. In general, however, the genomic profile of relapsed SCLC was very similar to that found before treatment, suggesting non-genetic mechanisms of acquired resistance, although the limited number of genes interrogated by our NGS panel does not allow us to draw firm conclusions.

The main limitation of the present study is the small sample size, which makes it difficult to deepen some biological issues, such as the correlation of circulating biomarkers with tissue genomic profiling and respective molecular subgroups. However, the study was exploratory in nature, and the prospective nature of the data and samples collection lays the groundwork for a validation multicenter prospective study in a real-world population receiving first line chemoimmunotherapy, as well as in randomized clinical trials where cfDNA, TF and tumor-related VAF may be considered as stratification biomarkers for intensification strategies.

## Conclusions

Baseline and dynamic changes of cfDNA, TF and VAF may help physicians to stratify ES-SCLC patients receiving first-line ACE and to anticipate the clinical course of the disease. Monitoring these biomarkers throughout therapy could not only provide useful insights into treatment response but also enable an early assessment of disease progression. Specifically, analyzing their temporal variations may enhance the ability to predict the clinical course of the disease, facilitating more personalized therapeutic decisions and potentially improving treatment outcomes.

## Electronic supplementary material

Below is the link to the electronic supplementary material.

Supplementary Figures and Table

## Data Availability

The datasets generated and during the current study are available in the Sequence Read Archive (SRA) repository under accession number PRJNA1260986.

## Abbreviations

A: Atezolizumab
A+: ASCL
AUC: Intravenous Administration of Carboplatin
CE: Carboplatin-Etoposide
cfDNA: cell-free DNA
CI: Confidence Interval
CNS: Central Nervous System
CTCAE: Common Toxicity Criteria for Adverse Events
ctDNA: circulating tumor DNA
ES: Extensive Stage
GEP: Gene Expression Profiling
HR: Hazard Ratios
I: Inflamed
IQRs: Interquartile Ranges
ITT: Intention-To-Treat
M: Median
MTB: Tumor Mutational Burden
N: Neuroendocrine
NGS: Next Generation Sequencing
OS: Overall Survival
PD-L1: Programmed Death-Ligand 1
PFS: Progression Free Survival
PH: Proportional Hazards
RECIST: Response Evaluation Criteria in Solid Tumors
SCLC: Small Cell Lung Cancer
TF: Tumor Fraction
TME: Tumor Microenvironment
VAF: Variant Allele Frequency
VIMP: Variable Permutation Importance Measure
VUS: Variant of Uncertain/Unknown Significance
sWGS: shallow Whole-Genome Sequencing

## References

[1] Rudin C, Brambilla E, Faivre-Finn C, Sage J. Small-cell lung cancer. Nat Rev Dis Prim. 10.1038/s41572-020-00235-0 10.1038/s41572-020-00235-0 PMC8177722 33446664

[2] Govindan R, Page N, Morgensztern D, et al. Changing epidemiology of small-cell lung cancer in the united States over the last 30 years: analysis of the surveillance, epidemiologic, and end results database. J Clin Oncol Off J Am Soc Clin Oncol. 2006;24(28):4539–44. 10.1200/JCO.2005.04.4859. 10.1200/JCO.2005.04.4859 17008692

[3] Mascaux C, Paesmans M, Berghmans T, et al. A systematic review of the role of Etoposide and cisplatin in the chemotherapy of small cell lung cancer with methodology assessment and meta-analysis. Lung Cancer. 2000;30(1):23–36. 10.1016/s0169-5002(00)00127-6. 11008007 10.1016/s0169-5002(00)00127-6

[4] Paz-Ares L, Dvorkin M, Chen Y, et al. Durvalumab plus platinum-etoposide versus platinum-etoposide in first-line treatment of extensive-stage small-cell lung cancer (CASPIAN): a randomised, controlled, open-label, phase 3 trial. Lancet (London England). 2019;394(10212):1929–39. 10.1016/S0140-6736(19)32222-6. 31590988 10.1016/S0140-6736(19)32222-6

[5] Horn L, Mansfield AS, Szczęsna A, et al. First-Line Atezolizumab plus chemotherapy in Extensive-Stage Small-Cell lung Cancer. N Engl J Med. 2018;379(23):2220–9. 10.1056/NEJMoa1809064. 30280641 10.1056/NEJMoa1809064

[6] Rudin CM, Awad MM, Navarro A, et al. Pembrolizumab or placebo plus Etoposide and platinum as First-Line therapy for Extensive-Stage Small-Cell lung cancer: randomized, Double-Blind, phase III KEYNOTE-604 study. J Clin Oncol Off J Am Soc Clin Oncol. 2020;38(21):2369–79. 10.1200/JCO.20.00793. 10.1200/JCO.20.00793 PMC7474472 32468956

[7] Wang J, Zhou C, Yao W, et al. Adebrelimab or placebo plus carboplatin and Etoposide as first-line treatment for extensive-stage small-cell lung cancer (CAPSTONE-1): a multicentre, randomised, double-blind, placebo-controlled, phase 3 trial. Lancet Oncol. 2022;23(6):739–47. 10.1016/S1470-2045(22)00224-8. 35576956 10.1016/S1470-2045(22)00224-8

[8] Cheng Y, Han L, Wu L, et al. Effect of First-Line Serplulimab vs placebo added to chemotherapy on survival in patients with Extensive-Stage small cell lung cancer: the ASTRUM-005 randomized clinical trial. JAMA. 2022;328(12):1223–32. 10.1001/jama.2022.16464. 36166026 10.1001/jama.2022.16464 PMC9516323

[9] Reck M, Dziadziuszko R, Sugawara S, et al. Five-year survival in patients with extensive-stage small cell lung cancer treated with Atezolizumab in the phase III IMpower133 study and the phase III imbrella A extension study. Lung Cancer. 2024;196:107924. 10.1016/j.lungcan.2024.107924. 39306923 10.1016/j.lungcan.2024.107924

[10] Lorenzi M, Resi MV, Bonanno L, et al. Tissue and Circulating biomarkers of benefit to immunotherapy in extensive-stage small cell lung cancer patients. Front Immunol. 2024;15:1308109. 10.3389/fimmu.2024.1308109. 38348046 10.3389/fimmu.2024.1308109 PMC10859471

[11] Liu SV, Reck M, Mansfield AS, et al. Updated overall survival and PD-L1 subgroup analysis of patients with Extensive-Stage Small-Cell lung Cancer treated with atezolizumab, carboplatin, and Etoposide (IMpower133). J Clin Oncol Off J Am Soc Clin Oncol. 2021;39(6):619–30. 10.1200/JCO.20.01055. 10.1200/JCO.20.01055 PMC8078320 33439693

[12] Chung HC, Piha-Paul SA, Lopez-Martin J, et al. Pembrolizumab after two or more lines of previous therapy in patients with recurrent or metastatic SCLC: results from the KEYNOTE-028 and KEYNOTE-158 studies. J Thorac Oncol Off Publ Int Assoc Study Lung Cancer. 2020;15(4):618–27. 10.1016/j.jtho.2019.12.109. 10.1016/j.jtho.2019.12.109 31870883

[13] Gadgeel SM, Pennell NA, Fidler MJ, et al. Phase II study of maintenance pembrolizumab in patients with Extensive-Stage small cell lung Cancer (SCLC). J Thorac Oncol Off Publ Int Assoc Study Lung Cancer. 2018;13(9):1393–9. 10.1016/j.jtho.2018.05.002. 10.1016/j.jtho.2018.05.002 PMC6833950 29775808

[14] Ott PA, Elez E, Hiret S, et al. Pembrolizumab in patients with Extensive-Stage Small-Cell lung cancer: results from the phase Ib KEYNOTE-028 study. J Clin Oncol Off J Am Soc Clin Oncol. 2017;35(34):3823–9. 10.1200/JCO.2017.72.5069. 10.1200/JCO.2017.72.5069 28813164

[15] Owonikoko TK, Park K, Govindan R, et al. Nivolumab and ipilimumab as maintenance therapy in Extensive-Disease Small-Cell lung cancer: checkmate 451. J Clin Oncol Off J Am Soc Clin Oncol. 2021;39(12):1349–59. 10.1200/JCO.20.02212. 10.1200/JCO.20.02212 PMC8078251 33683919

[16] Hellmann MD, Callahan MK, Awad MM, et al. Tumor mutational burden and efficacy of nivolumab monotherapy and in combination with ipilimumab in Small-Cell lung Cancer. Cancer Cell. 2018;33(5):853–e8614. 10.1016/j.ccell.2018.04.001. 29731394 10.1016/j.ccell.2018.04.001 PMC6750707

[17] Tosi A, Lorenzi M, Del Bianco P, et al. Extensive-stage small-cell lung cancer in patients receiving Atezolizumab plus carboplatin-etoposide: stratification of outcome based on a composite score that combines gene expression profiling and immune characterization of microenvironment. J Immunother cancer. 2024;12(7). 10.1136/jitc-2024-008974. 10.1136/jitc-2024-008974 PMC11218000 38955418

[18] Gay CM, Stewart CA, Park EM, et al. Patterns of transcription factor programs and immune pathway activation define four major subtypes of SCLC with distinct therapeutic vulnerabilities. Cancer Cell. 2021;39(3):346–e3607. 10.1016/j.ccell.2020.12.014. 33482121 10.1016/j.ccell.2020.12.014 PMC8143037

[19] Seo J, Kumar M, Mason J, et al. Plasticity of Circulating tumor cells in small cell lung cancer. Sci Rep. 2023;13(1):11775. 10.1038/s41598-023-38881-5. 37479829 10.1038/s41598-023-38881-5 PMC10362013

[20] Zhang J, Tian C, Lv F, et al. Molecular analysis of cell-free DNA identifies distinct molecular features in patients with chemosensitive and chemorefractory small cell lung cancer. Cancer Commun (London England). 2019;39(1):20. 10.1186/s40880-019-0363-y. 10.1186/s40880-019-0363-y PMC6472086 30999954

[21] Mohan S, Foy V, Ayub M, et al. Profiling of Circulating free DNA using targeted and Genome-wide sequencing in patients with SCLC. J Thorac Oncol Off Publ Int Assoc Study Lung Cancer. 2020;15(2):216–30. 10.1016/j.jtho.2019.10.007. 10.1016/j.jtho.2019.10.007 PMC7001105 31629061

[22] Almodovar K, Iams WT, Meador CB, et al. Longitudinal cell-Free DNA analysis in patients with small cell lung Cancer reveals dynamic insights into treatment efficacy and disease relapse. J Thorac Oncol Off Publ Int Assoc Study Lung Cancer. 2018;13(1):112–23. 10.1016/j.jtho.2017.09.1951. 10.1016/j.jtho.2017.09.1951 PMC5827950 28951314

[23] Tosello V, Grassi A, Rose D, et al. Binary classification of copy number alteration profiles in liquid biopsy with potential clinical impact in advanced NSCLC. Sci Rep. 2024;14(1):18545. 10.1038/s41598-024-68229-6. 39122833 10.1038/s41598-024-68229-6 PMC11316016

[24] Potente S, Boscarino D, Paladin D, Marchini S, Beltrame L, Romualdi C. SAMURAI: shallow analysis of copy number alterations using a reproducible and integrated bioinformatics pipeline. Brief Bioinform. 2024;26(1). 10.1093/bib/bbaf035. 10.1093/bib/bbaf035 PMC11775468 39879385

[25] Adalsteinsson VA, Ha G, Freeman SS, et al. Scalable whole-exome sequencing of cell-free DNA reveals high concordance with metastatic tumors. Nat Commun. 2017;8(1):1324. 10.1038/s41467-017-00965-y. 29109393 10.1038/s41467-017-00965-y PMC5673918

[26] Hsieh FY, Lavori PW. Sample-size calculations for the Cox proportional hazards regression model with nonbinary covariates. Control Clin Trials. 2000;21(6):552–60. 10.1016/s0197-2456(00)00104-5. 11146149 10.1016/s0197-2456(00)00104-5

[27] Chan JM, Quintanal-Villalonga Á, Gao VR, et al. Signatures of plasticity, metastasis, and immunosuppression in an atlas of human small cell lung cancer. Cancer Cell. 2021;39(11):1479–e149618. 10.1016/j.ccell.2021.09.008. 34653364 10.1016/j.ccell.2021.09.008 PMC8628860

[28] Liu SV, Mok TSK, Nabet BY, et al. Clinical and molecular characterization of long-term survivors with extensive-stage small cell lung cancer treated with first-line Atezolizumab plus carboplatin and Etoposide. Lung Cancer. 2023;186:107418. 10.1016/j.lungcan.2023.107418. 37931445 10.1016/j.lungcan.2023.107418

[29] Xie M, Vuko M, Rodriguez-Canales J, et al. Molecular classification and biomarkers of outcome with immunotherapy in extensive-stage small-cell lung cancer: analyses of the CASPIAN phase 3 study. Mol Cancer. 2024;23(1):115. 10.1186/s12943-024-02014-x. 38811992 10.1186/s12943-024-02014-x PMC11137956

[30] Husain H, Pavlick DC, Fendler BJ, et al. Tumor fraction correlates with detection of actionable variants across > 23,000 Circulating tumor DNA samples. JCO Precis Oncol. 2022;6:e2200261. 10.1200/PO.22.00261. 36265119 10.1200/PO.22.00261 PMC9616642

[31] Pizzutilo EG, Pedrani M, Amatu A, et al. Liquid biopsy for small cell lung Cancer either de Novo or transformed: systematic review of different applications and Meta-Analysis. Cancers (Basel). 2021;13(9). 10.3390/cancers13092265. 10.3390/cancers13092265 PMC8125928 34066817

[32] Bonanno L, Dal Maso A, Pavan A, et al. Liquid biopsy and non-small cell lung cancer: are we looking at the tip of the iceberg? Br J Cancer. 2022;127(3):383–93. 10.1038/s41416-022-01777-8. 35264788 10.1038/s41416-022-01777-8 PMC9345955

[33] Stejskal P, Goodarzi H, Srovnal J, Hajdúch M, van ’t Veer LJ, Magbanua MJM. Circulating tumor nucleic acids: biology, release mechanisms, and clinical relevance. Mol Cancer. 2023;22(1):1–21. 10.1186/s12943-022-01710-w. 36681803 10.1186/s12943-022-01710-w PMC9862574

[34] Ma L, Guo H, Zhao Y, et al. Liquid biopsy in cancer current: status, challenges and future prospects. Signal Transduct Target Ther. 2024;9(1):336. 10.1038/s41392-024-02021-w. 39617822 10.1038/s41392-024-02021-w PMC11609310

[35] Tsui DWY, Cheng ML, Shady M, et al. Tumor fraction-guided cell-free DNA profiling in metastatic solid tumor patients. Genome Med. 2021;13(1):96. 10.1186/s13073-021-00898-8. 34059130 10.1186/s13073-021-00898-8 PMC8165771

[36] Reichert ZR, Morgan TM, Li G, et al. Prognostic value of plasma Circulating tumor DNA fraction across four common cancer types: a real-world outcomes study. Ann Oncol Off J Eur Soc Med Oncol. 2023;34(1):111–20. 10.1016/j.annonc.2022.09.163. 10.1016/j.annonc.2022.09.163 PMC9805517 36208697

[37] Carbonell C, Frigola J, Pardo N, et al. Dynamic changes in Circulating tumor DNA assessed by shallow whole-genome sequencing associate with clinical efficacy of checkpoint inhibitors in NSCLC. Mol Oncol. 2023;17(5):779–91. 10.1002/1878-0261.13409. 36852704 10.1002/1878-0261.13409 PMC10158763

[38] Rolfo CD, Madison RW, Pasquina LW, et al. Measurement of ctdna tumor fraction identifies informative negative liquid biopsy results and informs value of tissue confirmation. Clin cancer Res Off J Am Assoc Cancer Res. 2024;30(11):2452–60. 10.1158/1078-0432.CCR-23-3321. 10.1158/1078-0432.CCR-23-3321 PMC11145175 38526394

[39] Malla M, Loree JM, Kasi PM, Parikh AR. Using Circulating tumor DNA in colorectal cancer: current and evolving practices. J Clin Oncol Off J Am Soc Clin Oncol. 2022;40(24):2846–57. 10.1200/JCO.21.02615. 10.1200/JCO.21.02615 PMC9390824 35839443

[40] Zulato E, Tosello V, Nardo G, Bonanno L, Del Bianco P, Indraccolo S. Implementation of next generation Sequencing-Based liquid biopsy for clinical molecular diagnostics in Non-Small cell lung Cancer (NSCLC) patients. Diagnostics (Basel Switzerland). 2021;11(8). 10.3390/diagnostics11081468. 10.3390/diagnostics11081468 PMC8394370 34441402

[41] Boscolo Bragadin A, Del Bianco P, Zulato E, et al. Longitudinal liquid biopsy predicts clinical benefit from immunotherapy in advanced non-small cell lung cancer. NPJ Precis Oncol. 2024;8(1):234. 10.1038/s41698-024-00704-9. 39420036 10.1038/s41698-024-00704-9 PMC11486993

[42] Galant N, Nicoś M, Kuźnar-Kamińska B, Krawczyk P. Variant allele frequency analysis of Circulating tumor DNA as a promising tool in assessing the effectiveness of treatment in Non-Small cell lung carcinoma patients. Cancers (Basel). 2024;16(4). 10.3390/cancers16040782. 10.3390/cancers16040782 PMC10887123 38398173

[43] Gao R, Lou N, Li L, et al. Mutational variant allele frequency profile as a biomarker of response to immune checkpoint Blockade in non-small cell lung Cancer. J Transl Med. 2024;22(1):576. 10.1186/s12967-024-05400-7. 38890738 10.1186/s12967-024-05400-7 PMC11184775

[44] Simons MJHG, Uyl-de Groot CA, Retèl VP, et al. Cost-Effectiveness and budget impact of future developments with Whole-Genome sequencing for patients with lung Cancer. Value Heal J Int Soc Pharmacoeconomics Outcomes Res. 2023;26(1):71–80. 10.1016/j.jval.2022.07.006. 10.1016/j.jval.2022.07.006 35973926

[45] Sivakumar S, Moore JA, Montesion M, et al. Integrative analysis of a large Real-World cohort of small cell lung Cancer identifies distinct genetic subtypes and insights into histologic transformation. Cancer Discov. 2023;13(7):1572–91. 10.1158/2159-8290.CD-22-0620. 37062002 10.1158/2159-8290.CD-22-0620 PMC10326603

