# Supplementary material for: Baseline levels and dynamic changes of cfDNA, tumor fraction and mutations to anticipate the clinical course of small cell lung cancer (SCLC) patients treated with first-line atezolizumab and chemotherapy: an hypothesis generating study (CATS/ML43257)
Source: J Exp Clin Cancer Res. 2025 Jun 19;44:178. doi: 10.1186/s13046-025-03434-3 (PMC12178010; doi:10.1186/s13046-025-03434-3)
Supplement: Supplementary file 1 — Supplementary Figures and Table [file 13046_2025_3434_MOESM1_ESM.pdf]

Supplementary figure 1: linear relationship between biomarker changes and hazard ratio for PFS (A-F) and OS (G-L)

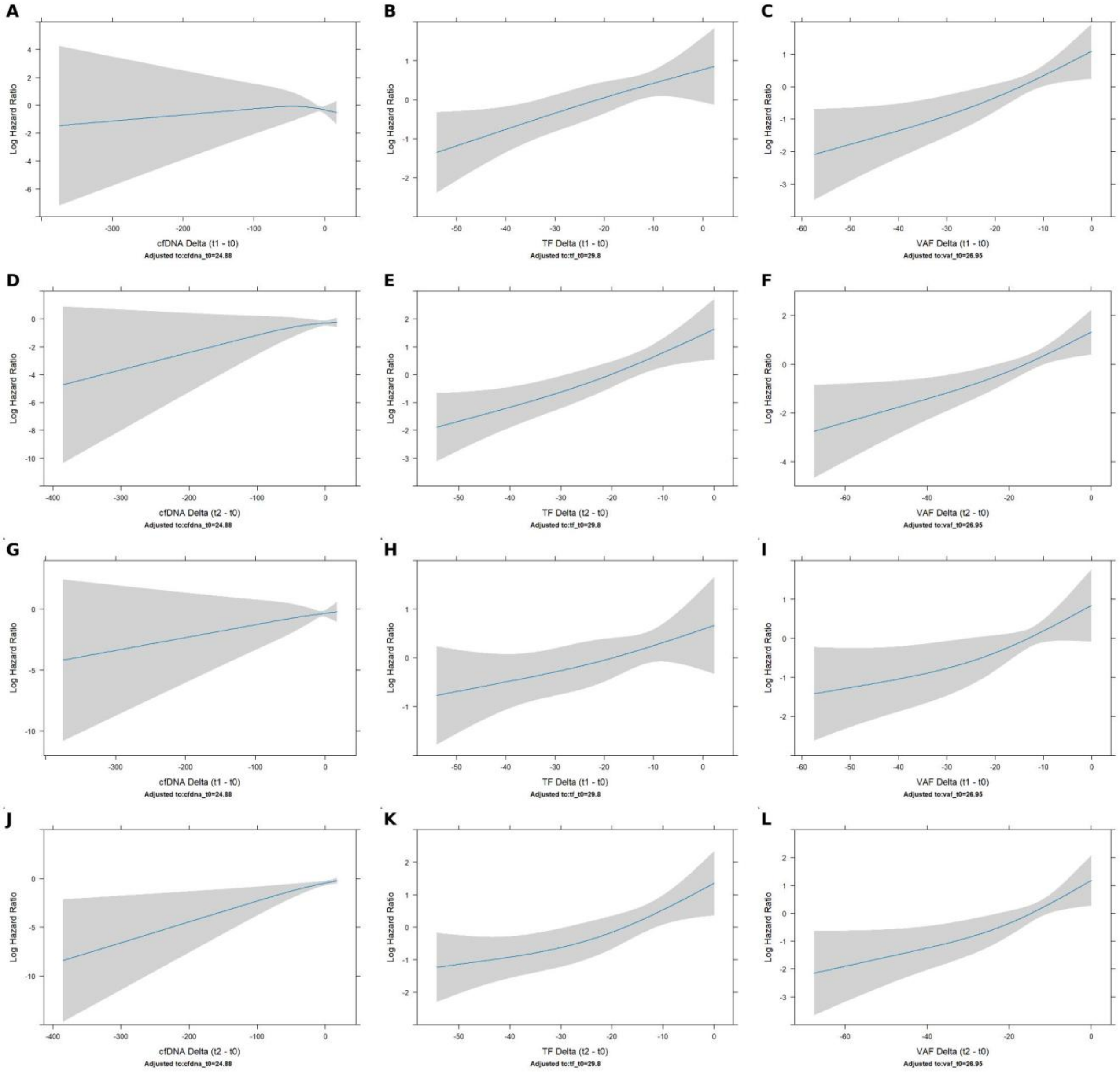

Supplementary figure 2: Kaplan-Meier curves of overall (OS) and progression free survival (PFS) in the intention to treat (ITT) population

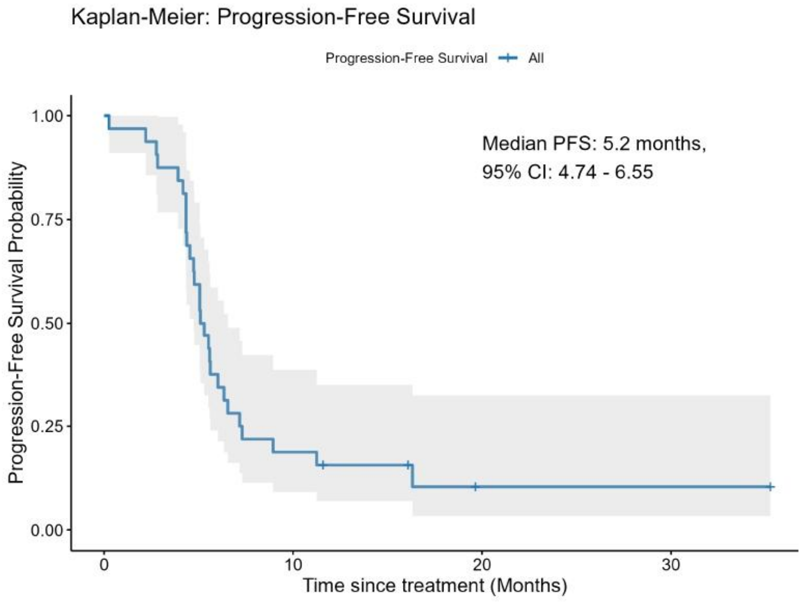

A.

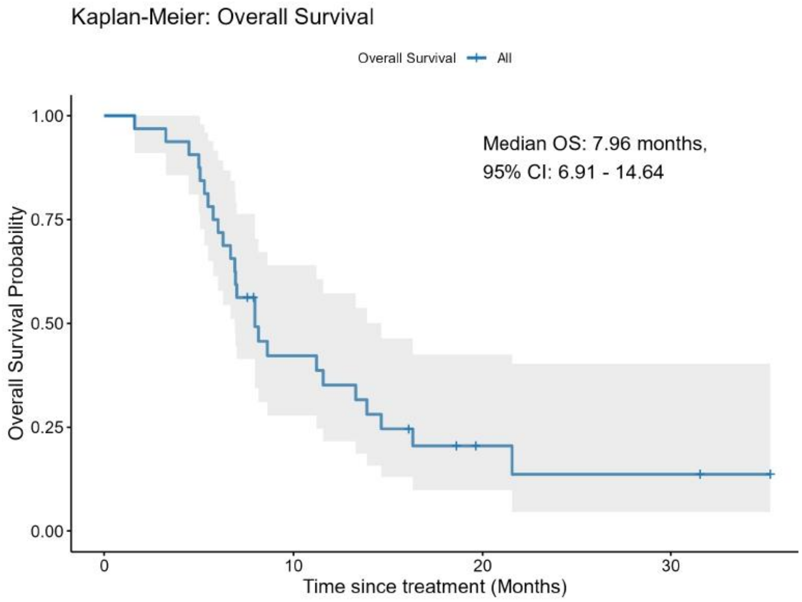

B.

Supplementary figure 3: Tumor fraction (TF) dynamics across timepoints (T0–T3) among patients with positive baseline TF.

Group 1 (blue circles) includes 20 patients (62.5%) with significant TF reductions at T1 or T2; group 2 (red squares) comprises 7 patients (21.9%) with minimal or no TF reductions at T1 and T2; Group 3 (green triangles) represents 5 patients (15.6%) with TF=0.00% at T0. Each data point represents the individual patient's TF (%), lines indicate the mean TF trend for each group across timepoints.

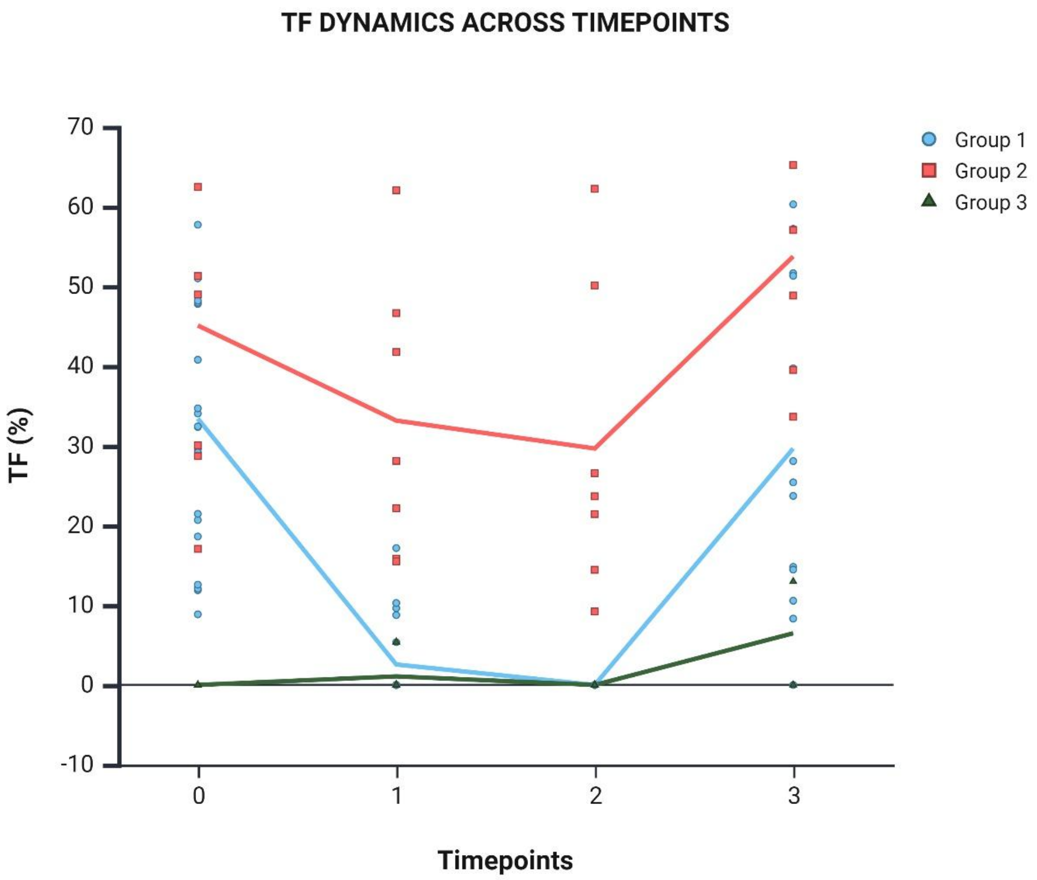

Supplementary table 1. Detailed Tumor Fraction values for each time point.

Sample	TF (%) T0	TF (%) T1	TF (%) T2	TF (%) T3
CATS-001	40.79	0	0	NA
CATS-002	62.46	46.64	26.56	78.27
CATS-003	20.69	0	0	NA
CATS-004	29.55	0	0	28.08
CATS-005	32.35	0	0	39.71
CATS-006	18.62	0	0	10.56
CATS-007	8.86	0	0	14.81
CATS-008	77.03	62.05	62.23	65.21
CATS-009	17.07	22.15	21.44	33.64
CATS-010	34.05	0	NA	NA
CATS-011	30.04	28.11	23.66	39.51
CATS-012	0	0	0	NA
CATS-013	32.42	8.77	0	NA
CATS-014	76.41	17.17	0	60.28
CATS-015	11.88	0	0	57.2
CATS-016	12.08	0	0	0
CATS-017	47.79	0	0	25.41
CATS-018	47.96	9.63	0	23.71
CATS-019	0	5.36	0	NA
CATS-020	57.72	0	0	NA
CATS-021	21.46	0	0	8.32
CATS-022	51	0	0	51.64
CATS-023	28.71	15.84	14.44	48.84
CATS-024	12.58	0	0	NA
CATS-025	29.17	5.37	0	14.47
CATS-026	48.2	10.28	0	NA
CATS-027	51.29	41.76	50.11	NA
CATS-029	34.7	0	0	51.32
CATS-030	0	0	0	12.97
CATS-031	0	0	0	0
CATS-032	48.97	15.49	9.206	57.07
CATS-033	0	0	0	NA
